# Impact of *Candida railenensis* during fermentation on the aromatic profile of Vidal blanc icewine

**DOI:** 10.3389/fmicb.2023.1192006

**Published:** 2023-08-08

**Authors:** Jing Li, Mengnan Hong

**Affiliations:** ^1^School of Food and Health, Jinzhou Medical University, Jinzhou, Liaoning, China; ^2^School of Life Science and Biotechnology, Dalian University of Technology, Dalian, Liaoning, China; ^3^Lab of Brewing Microbiology and Applied Enzymology, School of Biotechnology, Jiangnan University, Wuxi, Jiangsu, China

**Keywords:** *Candida railenensis*, *Saccharomyces cerevisiae*, sequential inoculation, mixed culture fermentation, Vidal blanc icewine, volatile aroma compounds

## Abstract

Mixed culture fermentation with non-*Saccharomyces* yeasts and *Saccharomyces cerevisiae* as multi-starters has more advantages than spontaneous fermentation, and wine products with distinctive and pleasant aromas can fulfill the diverse demands of consumers. This study was carried out to illuminate the effect of sequential inoculation of indigenous *Candida railenensis* and *S. cerevisiae* on alcoholic fermentation behavior and chemical and aromatic characteristics of Vidal blanc icewine. During the mixed culture fermentation, *C. railenensis* was present in the initial and middle stages but was absent after 14 days. The results of basic chemical parameters showed that the glycerol content in the mixed culture-fermented icewine was higher than that of the pure fermented icewine, but the acetic acid content was the opposite. In terms of volatile aroma compounds, *C. railenensis* in the mixed culture fermentation reduced some metabolites such as lower alcohols, 1-hexanol, 3-methylthiopropanol, and their unpleasant notes and increased the production of some desired volatile aroma compounds such as benzaldehyde, β-damascenone, 2-furanmethanol, and 5-methyl furfural associated with rose, honey, nut, and caramel characteristics. Furthermore, *C. railenensis* also changed the sensory performance of icewine by participating in the fermentation of *S. cerevisiae*. These findings suggest that *C. railenensis* with positive enological properties has the ability to be used in icewine production, which has never been reported before.

## 1. Introduction

Icewine is a typical dessert wine, which is made from ice grapes that are naturally frozen on the grapevine, and ice grapes must be harvested and pressed below −7°C (OIV-International Organisation of Vine Wine, 2015). Ice grape juice generally contains high concentrations of sugars (soluble solids level >35°Bx). The finished icewine is characterized by high levels of residual sugar (>125 g/L), titratable acid (>6.5 g/L, represented as tartaric acid), and aromatic compounds (Bowen and Reynolds, 2015). Because of the environmental and climatic requirements of ice grape growth and icewine production icewine can only be produced in a few countries such as Canada, Germany, Austria, and China (Alessandria et al., 2013). In China, the icewine industry has developed rapidly in recent years, with an annual output of approximately 3,000,000 L (Lan et al., 2019). Huanren is the major icewine-producing region in the northeast of China, and Vidal blanc is the main icewine grape cultivar in this region. Vidal blanc has high resistance to harsh climates, and its icewine has an appealing aroma and attractive flavor (Crandles et al., 2015).

Wine fermentation is a complex biochemical process in which the microbiota changes dynamically. In this process, yeast plays an important role, and yeast community succession occurs (Ballester-Tomás et al., 2017). Some studies have found that spontaneous fermentation is mainly triggered by non-*Saccharomyces* yeasts, which are then taken over by *Saccharomyces* spp. (mainly *S. cerevisiae*) and non-*Saccharomyces* yeasts that are more resistant to ethanol, such as *Candida* (Varela and Borneman, 2017; Li et al., 2019). Grape juice is converted into wine as a consequence of the combined effects of both *Saccharomyces* and non-*Saccharomyces* yeasts, and the sensory quality of the resultant wine depends on these effects (Li et al., 2018). Reportedly, some non-*Saccharomyces* yeasts are beneficial in improving the wine flavor and aroma complexity (Steensels and Verstrepen, 2014; Padilla et al., 2016; Binati et al., 2020) by secreting certain enzymes (such as esterases, β-glucosidase, and β-xylosidase) (Maturano et al., 2012; Hong et al., 2019). In recent years, the application of multi-starters of non-*Saccharomyces* yeast species together with *S. cerevisiae* in wine fermentation has been considered to satisfy consumer demands for wines in increasingly aromatic complexity (Englezos et al., 2018; Zhang et al., 2018; Lin et al., 2020). Several commercial non-*Saccharomyces* yeasts have emerged for mixed culture fermentation, such as *Lachancea thermotolerans, Metschnikowia pulcherrima, Pichia kluyveri, Schizosaccharomyces pombe*, and *Torulaspora delbrueckii* (Van Wyk et al., 2020).

Among non-*Saccharomyces* yeasts, some *Candida* species have been recognized to be able to tolerate wine-producing conditions and can be used in mixed culture fermentation together with *S. cerevisiae*, such as *Candida californica, Candida cantarellii, Candida pulcherrima, Candida sake*, and *Candida stellata* (Ciani et al., 2010; Rodríguez et al., 2010; Ballester-Tomás et al., 2017; Aplin et al., 2019; Hong et al., 2021). Among *Candida* species, the use of *Candida zemplinina* (synonym *Starmerella bacillaris*) in wine fermentation has been paid the most attention. *C. zemplinina* has been shown to improve the aroma profile of the wine, compared to single inoculation with *S. cerevisiae*, and it can increase the production of glycerol and reduce the production of acetic acid and volatile fatty acids, which are thought to have negative effects on wine quality (Rantsiou et al., 2012; Giaramida et al., 2013; Englezos et al., 2018; Tofalo et al., 2022).

With regard to *C. railenensis* associated with wine and grapes, recent studies have found it in some wine-producing regions (Brysch-Herzberg and Seidel, 2015; Drumonde-Neves et al., 2017); it has been also present in the early or even middle stages of spontaneous fermentation of wine (BreŽná et al., 2010; Cioch-Skoneczny et al., 2018; Li et al., 2018); and it has been detected to have satisfactory wine aroma-related enzyme activities (Hong et al., 2019; Lee and Park, 2020). Moreover, there has only been one initial attempt to use *C. railenensis* in wine fermentation, but little is known about it (Van Wyk et al., 2020), and so far, the research on the use of *C. railenensis* in icewine fermentation has not been found. The fermentation condition of icewine is different from that of general wine, which has the characteristics of high sugar concentration, high acid concentration, and low fermentation temperature (Zhang et al., 2018). Furthermore, there is no evidence that *C. railenensis* is pathogenic.

In our previous studies, indigenous *C. railenensis* strains were isolated from the spontaneous fermentation of Vidal blanc icewine (Li et al., 2018, 2019), and then, some *C. railenensis* strains were screened to have the potential to produce characteristic icewine through the experiments of tolerance and fermentation and aroma-related enzyme activities (β-glucosidases and β-xylosidase) (Hong et al., 2019). In this study, these *C. railenensis* strains were considered for use in icewine fermentation, based on the abilities of indigenous *C. railenensis* strains to adapt to the icewine micro-environment and their fermentation performance.

In terms of the strategy of mixed culture fermentation, many studies have proven that compared with simultaneous inoculation, sequential inoculation (especially *S. cerevisiae* was inoculated after 48 h of non-*Saccharomyces* yeast strains) could increase the production of glycerol in wine and achieve higher aroma complexity (Englezos et al., 2016; Lencioni et al., 2016; Zhang et al., 2018).

This study aimed to explore the possibility of *the C. railenensis* strain isolated from icewine being applied in icewine fermentation and its effect on the volatile profile of icewine. For this purpose, single *S. cerevisiae* fermentation and mixed culture fermentation of indigenous *C. railenensis* together with *S. cerevisiae* were carried out. The cell's growth was monitored during fermentation, and the basic chemical compositions and volatile aroma compounds of icewine produced by different fermentation strategies were also determined and compared under laboratory-scale conditions.

## 2. Materials and methods

### 2.1. Yeast strains

The yeast strains used in this study were isolated from the spontaneous fermentation of Vidal blanc icewine and identified by WL nutrient agar medium and the internal transcribed spacer (ITS) region sequences (Li et al., 2018). Then, a series of tolerance experiments (alcohol, sugar, SO_2_, and tartaric acid), fermentation experiments, and enzyme activity experiments (β-glucosidases, β-xylosidase, and pectinase) were carried out to screen for the indigenous strains of *S. cerevisiae* and *C. railenensis* (Hong et al., 2019). Finally, the indigenous strains (one strain of *S. cerevisiae* and one strain of *C. railenensis*) with the best comprehensive performance were screened for subsequent fermentation experiments. The *S. cerevisiae* strain had excellent alcoholic fermentation ability, and the *C. railenensis* strain had satisfactory cell wall-bound and extracellular enzyme activities among all non-*Saccharomyces* yeast strains studied (Hong et al., 2019). Table 1 provides basic information on the indigenous yeast strains (Li et al., 2018).

**Table 1:** The basic information of the indigenous yeast strains used in this study.

**Species**	**GenBank accession No. (ITS region)**	**Glucose^a^ (g/L)**	**Tartaric acid^a^ (g/L)**	**Ethanol^a^ (%)**	**SO${}_{2}^{\text{a}}$ (mg/L)**
*C. railenensis*	MH023198	>500	>20	>4, and < 8	>350
*S. cerevisiae*	MH006564	>500	>20	>10, and < 12	>350

a The tolerance results of the screened strains.

### 2.2. Fermentation trials

The ice grape juice of Vidal blanc used for icewine fermentation was obtained from the vineyard (Wunushan Milan Winery; NE China, 41°17′53.53″N 125°22′27.26″E) in Huanren Manchu Autonomous County, which is the most important icewine-producing region of China. It had a pH of 3.12, soluble solid content of 39.1 °Bx, and total acid and sugar contents of 5.12 g/L and 392.64 g/L, respectively.

Ice grape juice was heated at 70°C for 20 min to achieve sterilization (Maturano et al., 2012). Each 600 ml of the sterilized ice grape juice (adding 50 mg/L SO_2_) was in a 1-L sterile flask with a sterile glass air lock. The glass air lock contained concentrated sulfuric acid, which could allow CO_2_ produced during icewine fermentation to escape. Laboratory-inoculated fermentations were carried out at 18°C for 30 days. The indigenous strains of *S. cerevisiae* and *C. railenensis* were activated in the YPD (yeast extract 10 g/L, peptone 20 g/L, and dextrose 20 g/L; Haibo, Qingdao, China) medium. Different inoculated fermentation strategies were as follows: (1) pure culture fermentation (designated as S2): only *S. cerevisiae* was added to the sterilized ice grape juice (initial inoculum of approximately 10^6^ cells/ml final concentration); (2) mixed culture fermentation (designated as MF2S2): *C. railenensis* was added first (initial inoculum of approximately 10^6^ cells/ml final concentration), 48 h later, *S. cerevisiae* was also added to the sterilized ice grape juice; the addition ratio of *C. railenensis* and *S. cerevisiae* was approximately 1: 1.

### 2.3. Yeast population dynamic changes in icewine fermentation

Samplings were carried out at 0, 2, 4, 7, 14, 21, and 30 days during icewine fermentation. Before sampling, the flasks were shaken. Each sample was serially diluted (1, 10^4^ to 1, 10^6^ ratios) in a sterile physiological solution and spread-plated on WL nutrient agar medium, and all plates were incubated at 28°C for 5 days. The colonies of *C. railenensis* and *S. cerevisiae* on the WL nutrient agar medium could be differentiated (Li et al., 2018), and the number of colonies was counted and recorded.

### 2.4. Basic chemical composition analysis

All fermentation flasks were weighed every day during icewine fermentation, and the daily production of CO_2_ under different fermentation strategies was recorded. Fermentation was deemed finished when the weight of the flask remained constant for 2 consecutive days (Lencioni et al., 2016). The basic chemical compositions, such as total sugar and total acid contents, were measured, and the methods used adhered to the inspection standards proposed by the OIV (2018).

Ethanol content was determined by a Gas Chromatography (GC) 9,790 plus with a flame ionization detector (Fuli Analytical Instruments Co., Zhejiang, China). The chromatograph was equipped with a KB-5 capillary analytical column (30 m × 320 μm × 0.25 μm; Kromat Co., Bordentown, United States). Ethanol was used for apparatus calibration as a standard, and 1-propanol (Aladdin Biochemical Technology Co., Shanghai, China) was used as an internal standard. The column oven temperature was initially held at 45°C for 5 min, then increased by 5°C/min to 50°C and held for 5 min, and finally increased to 230°C at 20°C/min and held for 2 min. The injector and detector temperatures were 250°C. The carrier gas was nitrogen at a constant flow rate of 1 ml/min, and the injection split ratio was 50:1, and the injection volume was 1 μl.

Organic acid contents were determined by using a Prominence LC-20A system (Shimadzu Co., Japan) for high-performance liquid chromatography (HPLC). The chromatograph was equipped with a Wondasil C18-WR column (4.6 mm × 150 mm, 5 μm; Shimadzu Co., Japan). The column temperature was 35°C, the mobile phase was acetonitrile and phosphoric acid (pH 2.0) with a ratio of 2:98, and the flow rate was 0.8 ml/min.

These basic chemical compositions of each icewine sample were determined in triplicate.

### 2.5. Analysis of major volatile aroma compounds

The metabolites of icewines under different fermentation strategies were further measured by headSpace solid phase microextraction gas chromatography-tandem time-of-flight mass spectrometry (HS-SPME-GC-TOFMS). Each icewine was sampled and determined six times. A 3 ml sample of icewine and 0.6 g of NaCl were placed in a 20 ml microextraction vial and then mixed with 5 μl internal standard solution (IS, 2-octanol, 10 mg/L stock in dH_2_O). All samples were analyzed by GC (7890B; Agilent Technologies Inc., California, United States) system coupled with a mass spectrometer (MS, 5977B; Agilent Technologies Inc., California, United States). The chromatograph was equipped with a DB-Wax column (30 m × 250 μm × 0.25 μm; Agilent Technologies Inc., California, United States). The column oven temperature program was as follows: the initial temperature was kept at 40°C for 4 min, then raised to 230°C at a rate of 5°C/min, and held for 5 min. The detailed instrument parameters are presented in Table 2.

**Table 2:** The instrument parameters of HS-SPME-GC-TOFMS.

**Item**	**Parameter**
Incubate temperature	60°C
Preheat time	15 min
Incubate time	30 min
Desorption time	4 min
Front inlet mode	Splitless Mode
Front inlet septum purge flow	3 mL/min
Carrier gas	Helium
Column flow	1 mL/min
Injection temperature	250°C
Transfer line temperature	250°C
Ion source temperature	230°C
Quad temperature	150°C
Electron energy	−70 eV
Mass range	20–500 m/z
Solvent delay	0 min

Chroma TOF software (V4.3X, LECO Co., USA) and NIST database were used for raw peak extraction, the data baseline filtering and calibration of the baseline, peak alignment, deconvolution analysis, peak identification, integration, and spectrum match of the peak area (Kind et al., 2009). Principal component analysis (PCA) and orthogonal partial least squares discriminant analysis (OPLS-DA) were carried out using SIMCA (V15.0.2, Umetrics Inc., Sweden). The selection principles of differential volatile aroma compounds were as follows: the *P*-value of the Student's *t-*test was <0.05, and the variable importance in the projection (VIP) of the first principal component in the OPLS-DA model was >1. Data of the differential volatile aroma compounds selected were calculated as Euclidean distance matrix and were taken for cluster analysis.

### 2.6. Sensory analysis

In total, 10 students (five male students and five female students, aged between 21 and 25 years old) with experience in sensory evaluation were selected for sensory analysis of the fermented icewine. The students were trained to identify and evaluate the reference standards, which were prepared by adding corresponding aroma standard from *Le nez du vin* (Jean Lenoir, Provence, France) to 10% v/v aqueous ethanol (pH 3.4) and diluted in series; the training took place twice a week for 3 months (Huang et al., 2018). Finally, a 30 ml sample of icewine was placed in a wine glass with a cover, and the icewines fermented by different fermentation strategies were not marked before the sensory analysis. The six-aspect aromas of “nutty”, “tropical fruity”, “apricot and peach”, “honey”, “caramel and roasted”, and “floral (rose)” were graded (0–5 score; the higher score showed the stronger aroma). The sensory analysis of icewine was performed twice, and the final scores of the aroma of six aspects were the average value.

## 3. Results

### 3.1. Dynamic changes of yeast growth during fermentation

**Figure 1:**
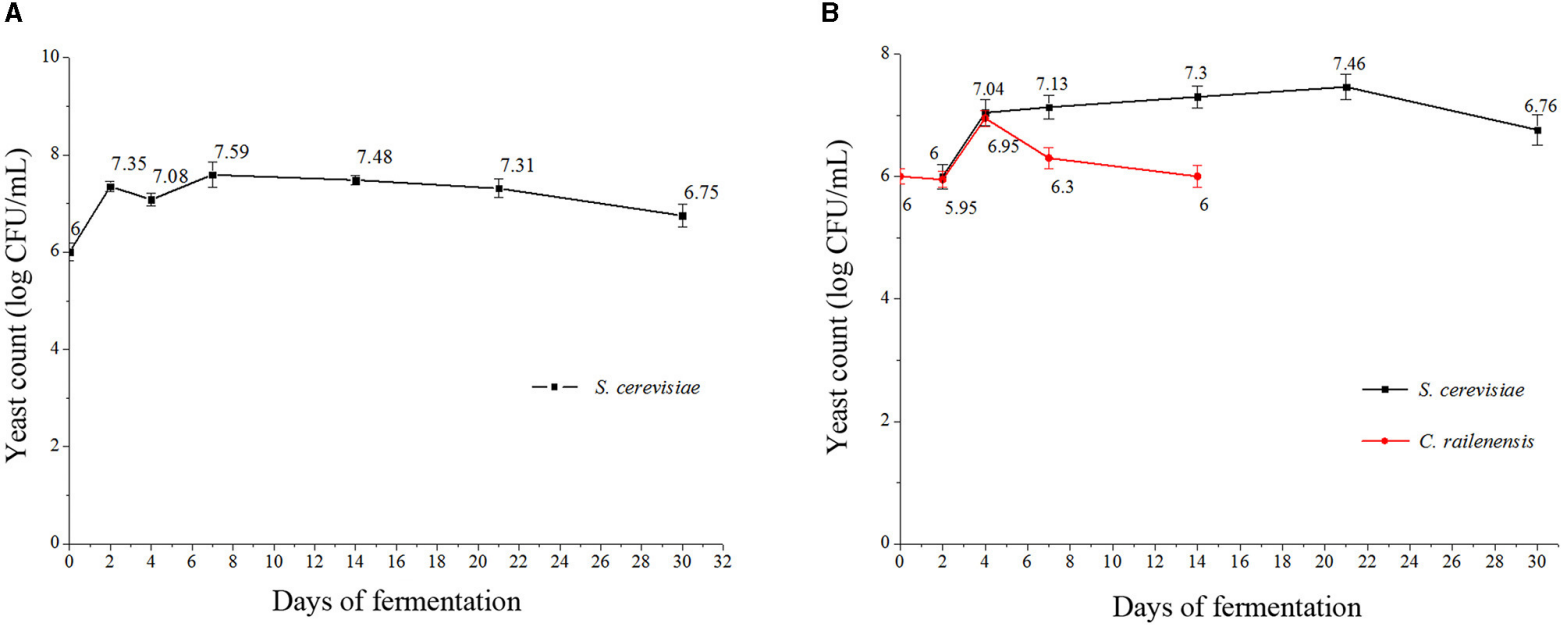
Cell growth dynamics of *S. cerevisiae* and *C. railenensis* during pure and mixed culture fermentation. **(A)** S2, pure culture fermentation; **(B)** MF2S2, mixed culture fermentation. Counts are the mean CFU/ml values ± standard deviations.

The yeast growth dynamics were detected by plate counting at 0, 2, 4, 7, 14, 21, and 30 days during the pure culture fermentation (S2) and the sequential mixed culture fermentation (MF2S2), and the results are presented in Figures 1A, B. In S2, the maximum cell population of *S. cerevisiae* was observed on day 7 (approximately 7.59 log CFU/ml) and then decreased. After 30 days, *S. cerevisiae* cells still presented good vitality, with approximately 6.75 log CFU/ml. In MF2S2, the cell population of *S. cerevisiae* first gradually increased and reached the highest value on day 21 (7.46 log CFU/ml) and then decreased. In terms of the cell population of *C. railenensis*, there was no obvious change in the first 2 days of MF2S2; after inoculating *S. cerevisiae*, the cell population of *C. railenensis* increased and reached the maximum population on day 4 (6.95 log CFU/ml). However, *C. railenensis* was not detected after 14 days, while *S. cerevisiae* maintained good viability (above 10^6^ CFU/ml) until the end of the monitored period.

### 3.2. Chemical parameters

#### 3.2.1. CO_2_ and ethanol production during fermentation

**Figure 2:**
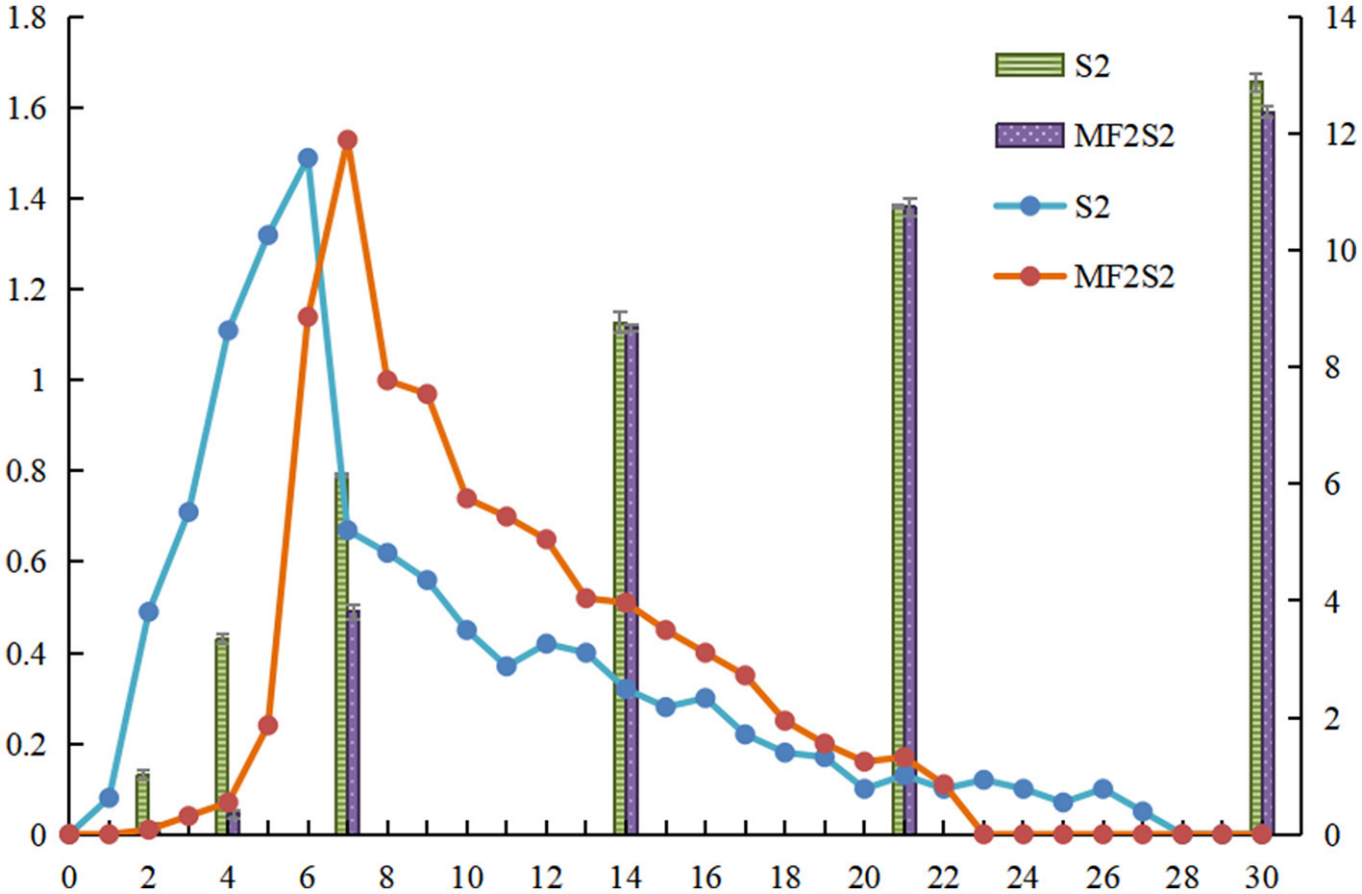
CO_2_ production and ethanol content during icewine fermentation. The column shows the ethanol content of icewine. The line shows CO_2_ production, and the standard deviation values are 0–0.02.

The daily production of CO_2_ and the ethanol content was measured during icewine fermentation, as shown in Figure 2. The maximum daily productions of CO_2_ during S2 and MF2S2 were observed on days 6 and 7 and were 1.49 g/L and 1.53 g/L, respectively. In the first 2 days of MF2S2, changes in CO_2_ production and ethanol contents were not obvious; after inoculating *S. cerevisiae*, the daily production of CO_2_ changes was not much from days 2 to 4 (from 0.01 g/L to 0.07 g/L), and the ethanol contents changed from 0.13% (v/v) to 0.35% (v/v). However, CO_2_ productions (from 0 to 1.01 g/L) and ethanol contents (from 0 to 3.35%, v/v) in the first 4 days of S2 were both obviously higher than those in MF2S2. At the end of S2 and MF2S2, the daily productions of CO_2_ for both were 0, indicating the fermentation had been completed, and the total productions of CO_2_ were 10.92 g/L and 10.22 g/L, respectively. Accordingly, ethanol concentration in icewine of S2 (12.89 ±0.08%, v/v) was higher than that in icewine of MF2S2 (12.37 ± 0.07%, v/v).

#### 3.2.2. Other basic chemical parameters in icewine

The concentrations of residual sugar, ethanol content, tartaric acid, acetic acid, succinic acid, and glycerol in the icewines fermented by pure and mixed culture fermentation are presented in Table 3. The contents of residual sugar, tartaric acid, succinic acid, and glycerol in the icewine of MF2S2 were all higher than those in the icewine of S2. The residual sugar contents in icewines of MF2S2 and S2 met the requirements of Vintners Quality Alliance (VQA) that icewine should be required to have a residual sugar content of >125 g/L (Bowen and Reynolds, 2015). Moreover, the glycerol content in icewine of MF2S2 was 11.18 ± 0.12 g/L, which was higher than that in icewine of S2 (10.91 ± 0.33 g/L), and they are both >10 g/L. The acetic acid content was 2.03 ± 0.13 g/L in icewine of S2 and 1.82 ± 0.17 g/L in icewine of MF2S2, both of which were lower than the maximum allowable limit in OIV (2.1 g/L).

**Table 3:** Final chemical parameters of icewines produced by pure and mixed culture fermentations.

**No**.	**Residul sugar (g/L)**	**Ethanol content (%, v/v)**	**Tartaric acid (g/L)**	**Acetic acid (g/L)**	**Succinic acid (g/L)**	**Glycerol (g/L)**
S2	158.61 ± 1.71	12.89 ± 0.08	5.53 ± 0.33	2.03 ± 0.13	1.49 ± 0.82	10.91 ± 0.33
MF2S2	181.21 ± 0.99	12.37 ± 0.07	6.17 ± 0.02	1.82 ± 0.17	1.55 ± 0.02	11.18 ± 0.12

### 3.3. Volatile aroma compounds

**Figure 3:**
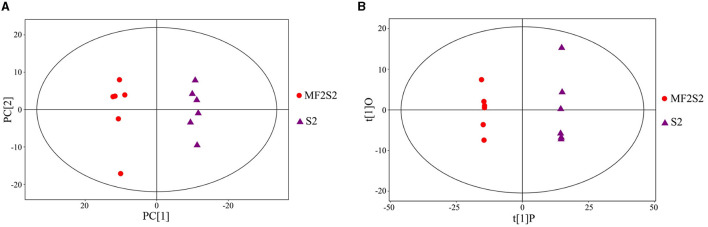
Scatter plots of PCA **(A)** and OPLS-DA **(B)** model for icewine samples of MF2S2 and S2.

The identification of the volatile aroma compounds in the icewines fermented by pure and mixed culture fermentation was carried out by HS-SPME-GC-TOFMS. The principal component analysis (PCA) scatter plot of the icewine samples of MF2S2 and S2 is shown in Figure 3A, and it was acquired that the R^2^X (cum) was 0.524. To achieve a higher level of group separation and obtain a better understanding of classification variables, orthogonal partial least squares-discrimination analysis (OPLS-DA) was also used, and the scatter plot is shown in Figure 3B; the parameters obtained were R^2^X (cum) = 0.481, R^2^Y (cum) = 1 and Q^2^ (cum) = 0.971, which were good and stable for prediction and fitness. In Figure 3A, the abscissa PC[1] and the ordinate PC[2] represent the scores of the first and second principal components; in Figure 3B, the abscissa t[1]P represents the score of the predicted first principal component, and the ordinate t[1]O represents the score of orthogonal principal component; the scatter dots of different colors and shapes represent the icewine samples with different fermentation strategies, as shown in Figures 3A, B. The score plots indicate that the difference between the two groups of icewine samples is very significant, and all of the samples are within the 95% confidence interval, which reflects high experimental reproducibility and the different patterns of volatile metabolites.

**Figure 4:**
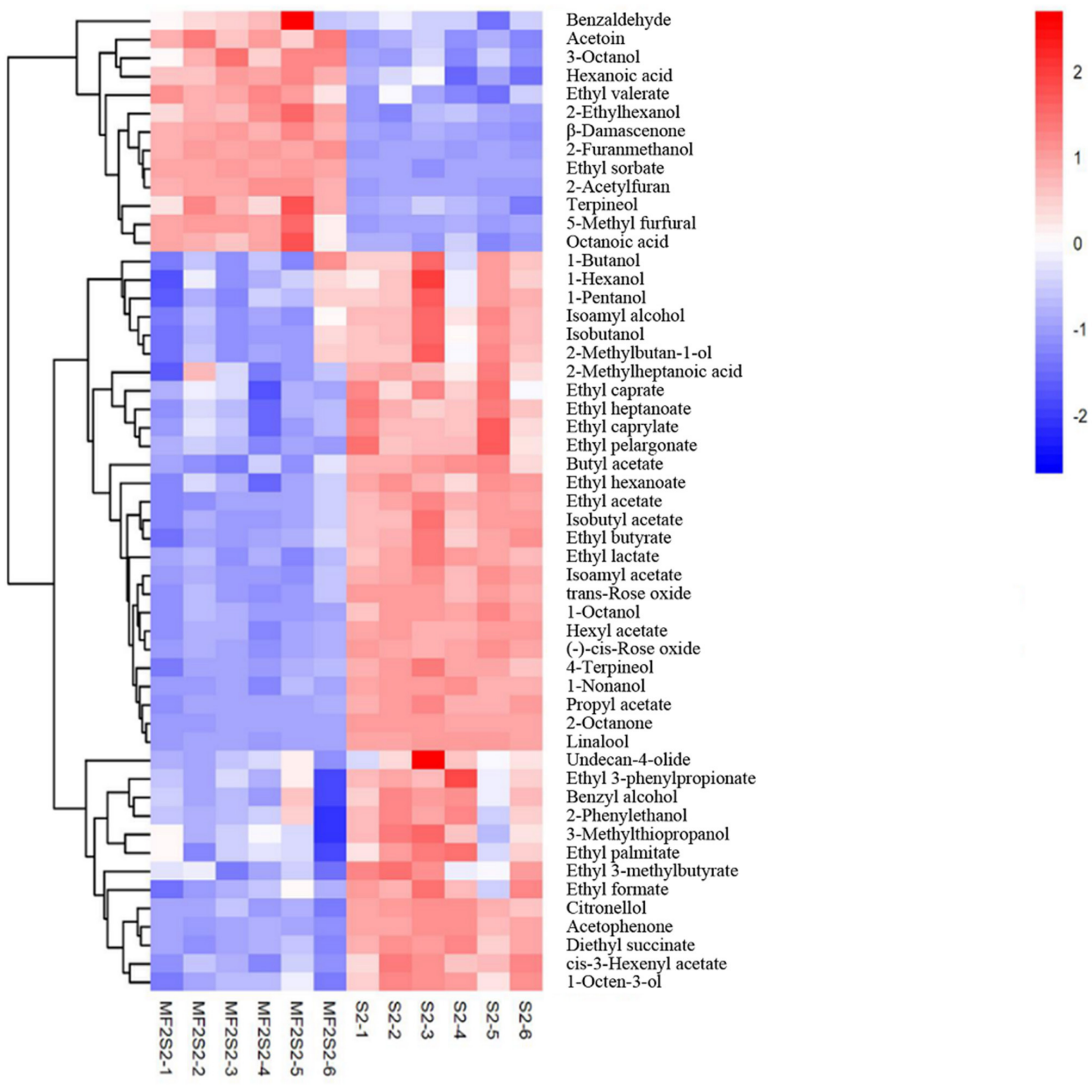
The heat map of volatile aroma compounds in icewines of MF2S2 and S2.

When the screening principle was *P* < 0.05 and VIP > 1, a total of 248 differential volatile metabolites were detected. The further screening principle was matching similarity of >600, and meanwhile, physical and chemical properties and aroma of different metabolites were also considered. In brief, 52 differential volatile aroma compounds in icewines of MF2S2 and S2 were screened and subsequently divided into 4 groups, including 20 esters, 15 alcohols, 7 terpenes, and 10 others, as shown in Table 4. These metabolites were also clustered and displayed by heat map (Figure 4).

**Table 4:** Comparison of volatile aroma compounds in the icewines fermented by pure and mixed culture fermentation.

**Volatile metabolites**	**Peak**	**CAS**	**Similarity**	**RT**	**Mass**	**MF2S2**	**S2**	**VIP**	***P*-Value**	**FC**
**Esters (20)**										
Ethyl acetate	Ethyl acetate	141-78-6	946	3.029	43	40.319 ± 0.912	56.643 ± 1.544	1.553	3.801E-08	0.712
Ethyl butyrate	Butanoic acid, ethyl ester	105-54-4	929	6.07	71	0.861 ± 0.02	0.991 ± 0.058	1.518	1.466E-06	0.869
Ethyl caprate	Decanoic acid, ethyl ester	110-38-3	971	22.558	88	13.601 ± 0.511	16.827 ± 0.687	1.323	6.499E-04	0.808
Ethyl caprylate	Octanoic acid, ethyl ester	110-38-3	966	17.509	88	8.205 ± 0.312	11.385 ± 0.454	1.433	5.283E-05	0.721
Ethyl formate	Ethyl formate	109-94-4	765	2.444	31	0.081 ± 0.004	0.098 ± 0.005	1.314	0.001	0.826
Ethyl hexanoate	Hexanoic acid, ethyl ester	123-66-0	978	11.873	88	7.617 ± 0.33	8.535 ± 0.454	1.482	7.542E-06	0.892
Ethyl heptanoate	Heptanoic acid, ethyl ester	106-30-9	918	14.739	88	0.177 ± 0.091	0.219 ± 0.052	1.455	2.372E-05	0.808
Ethyl lactate	Propanoic acid, 2-hydroxy-, ethyl ester	97-64-3	904	14.944	45	0.142 ± 0.072	0.187 ± 0.081	1.551	9.550E-08	0.757
Ethyl palmitate	Hexadecanoic acid, ethyl ester	628-97-7	905	35.058	88	0.090 ± 0.003	0.130 ± 0.055	1.128	7.139E-03	0.691
Ethyl pelargonate	Nonanoic acid, ethyl ester	123-29-5	862	20.071	88	0.030 ± 0.002	0.049 ± 0.002	1.484	2.167E-05	0.606
Ethyl valerate^*^	Pentanoic acid, ethyl ester	539-82-2	898	8.872	88	0.174 ± 0.051	0.156 ± 0.02	1.448	2.785E-05	1.114
Ethyl 3-methylbutyrate	Butanoic acid, 3-methyl-, ethyl ester	105019-18-9	653	26.248	87	0.814 ± 0.05	1.045 ± 0.21	1.298	1.431E-03	0.779
Ethyl 3-phenylpropionate	Benzenepropanoic acid, ethyl ester	2021-28-5	844	27.869	104	0.049 ± 0.001	0.061 ± 0.003	1.248	2.658E-03	0.799
Butyl acetate	Acetic acid, butyl ester	123-86-4	845	7.032	56	0.022 ± 0	0.026 ± 0.001	1.473	1.235E-05	0.839
Diethyl succinate	Butanedioic acid, diethyl ester	123-25-1	917	23.352	129	0.137 ± 0.013	0.239 ± 0.043	1.553	1.051E-07	0.574
Hexyl acetate	Acetic acid, hexyl ester	142-92-7	950	12.987	56	0.634 ± 0.014	0.816 ± 0.025	1.573	1.269E-09	0.778
cis-3-Hexenyl acetate	3-Hexen-1-ol, acetate, (Z)-	3681-71-8	891	14.229	67	0.112 ± 0.032	0.129 ± 0.035	1.501	7.156E-06	0.871
Isobutyl acetate	Isobutyl acetate	110-19-0	942	5.463	56	0.124 ± 0.03	0.156 ± 0.022	1.540	3.390E-07	0.795
Isoamyl acetate	1-Butanol, 3-methyl-, acetate	123-92-2	957	8.498	70	6.011 ± 0.285	8.592 ± 0.307	1.563	8.332E-09	0.700
Propyl acetate	n-Propyl acetate	109-60-4	888	4.59	61	0.050 ± 0	0.082 ± 0.002	1.585	1.745E-10	0.615
**Alcohols (15)**										
1-Butanol	1-Butanol	71-36-3	947	9.31	56	0.364 ± 0.053	0.399 ± 0.068	1.043	0.021	0.912
1-Pentanol	1-Pentanol	71-41-0	945	12.439	42	0.416 ± 0.075	0.456 ± 0.082	1.222	0.003	0.912
Isobutanol	1-Propanol, 2-methyl-	78-83-1	928	7.815	42	2.485 ± 0.56	2.866 ± 0.66	1.328	6.639E-04	0.867
Isoamyl alcohol	1-Butanol, 3-methyl-	123-51-3	934	11.272	55	30.942 ± 0.712	36.804 ± 0.85	1.402	1.391E-04	0.841
2-Methylbutan-1-ol	1-Butanol, 2-methyl-	137-32-6	893	11.19	56	13.384 ± 0.511	15.042 ± 0.558	1.260	0.002	0.890
1-Hexanol	1-Hexanol	111-27-3	958	15.362	56	2.532 ± 0.457	2.677 ± 0.512	1.118	0.011	0.946
1-Nonanol	1-Nonanol	143-08-8	828	23.091	56	0.064 ± 0.001	0.099 ± 0.002	1.578	3.562E-10	0.650
1-Octanol	1-Octanol	111-87-5	892	20.665	56	0.182 ± 0.043	0.245 ± 0.077	1.575	2.672E-09	0.744
3-Octanol^*^	3-Octanol	589-98-0	900	16.493	59	0.532 ± 0.092	0.487 ± 0.08	1.443	6.740E-05	1.094
2-Ethylhexanol^*^	1-Hexanol, 2-ethyl-	104-76-7	932	18.968	57	0.371 ± 0.059	0.310 ± 0.048	1.518	2.171E-06	1.196
1-Octen-3-ol	1-Octen-3-ol	3191-86-4	975	17.938	57	2.484 ± 0.54	2.668 ± 0.522	1.468	2.912E-05	0.931
Benzyl alcohol	Benzyl alcohol	100-51-6	949	27.701	107	0.133 ± 0.034	0.151 ± 0.04	1.229	0.004	0.879
2-Phenylethanol	Phenylethyl alcohol	60-12-8	960	28.448	91	24.984 ± 0.544	27.816 ± 0.844	1.130	0.010	0.898
3-Methylthiopropanol	1-Propanol, 3-(methylthio)-	505-10-2	850	24.284	61	0.021 ± 0	0.026 ± 0.001	1.036	0.020	0.818
2-Furanmethanol^*^	2-Furanmethanol	623-19-8	880	22.971	98	0.286 ± 0.044	0.084 ± 0.002	1.593	9.702E-09	3.388
**Terpenes (7)**										
Citronellol	Citronellol	68916-43-8	864	25.456	69	0.132 ± 0.024	0.176 ± 0.022	1.559	5.460E-08	0.746
Linalool	Linalool	78-70-6	941	20.37	93	0.332 ± 0.044	0.602 ± 0.084	1.592	3.051E-14	0.551
Terpineol^*^	Terpineol	8000-41-7	830	23.88	93	0.914 ± 0.088	0.812 ± 0.09	1.441	6.436E-05	1.126
4-Terpineol	3-Cyclohexen-1-ol, 4-methyl-1-(1-methylethyl)-, (R)-	562-74-3	914	21.66	93	0.439 ± 0.071	0.540 ± 0.08	1.554	7.239E-08	0.812
(-)-cis-Rose oxide	(2S,4R)-4-Methyl-2-(2-methylprop-1-en-1-yl)tetrahydro-2H-pyran	3033-23-6	911	15.219	69	0.301 ± 0.054	0.463 ± 0.069	1.580	6.832E-11	0.651
trans-Rose oxide	(2R,4R)-4-Methyl-2-(2-methylprop-1-en-1-yl)tetrahydro-2H-pyran	5258-11-7	893	15.621	83	0.037 ± 0.001	0.059 ± 0.001	1.563	4.215E-09	0.631
β-Damascenone^*^	2-Buten-1-one, 1-(2,6,6-trimethyl-1,3-cyclohexadien-1-yl)-, (E)-	23726-93-4	940	26.548	121	0.353 ± 0.044	0.202 ± 0.014	1.581	5.246E-10	1.745
**Others (10)**										
Hexanoic acid^*^	Hexanoic acid	142-62-1	907	27.366	60	3.357 ± 0.088	2.864 ± 0.744	1.419	6.246E-05	1.172
Octanoic acid^*^	Octanoic acid	124-07-2	943	31.716	60	2.816 ± 0.684	2.151 ± 0.544	1.467	3.136E-05	1.309
2-Methylheptanoic acid	2-Methylheptanoic acid	1188-02-9	624	16.261	74	0.020 ± 0.001	0.023 ± 0	1.201	0.004	0.872
Acetoin^*^	Acetoin	513-86-0	873	13.184	88	0.089 ± 0	0.071 ± 0.001	1.520	2.343E-06	1.259
2-Octanone	2-Octanone	111-13-7	751	13.29	58	0.035 ± 0.001	0.078 ± 0.002	1.593	4.554E-14	0.450
Acetophenone	Acetophenone	98-86-2	916	22.633	105	0.065 ± 0.002	0.110 ± 0.011	1.584	4.795E-10	0.585
2-Acetylfuran^*^	Ethanone, 1-(2-furanyl)-	1192-62-7	895	19.119	95	0.392 ± 0.054	0.160 ± 0.024	1.589	3.430E-12	2.443
Benzaldehyde^*^	Benzaldehyde	17901-93-8	903	19.533	105	0.091 ± 0.004	0.081 ± 0.001	1.002	0.030	1.116
Undecan-4-olide	2(3H)-Furanone, 5-heptyldihydro-	104-67-6	775	23.794	85	0.101 ± 0.027	0.107 ± 0.012	1.003	0.0353	0.949
5-Methyl furfural^*^	2-Furancarboxaldehyde, 5-methyl-	620-02-0	873	20.801	110	0.255 ± 0.05	0.080 ± 0.002	1.552	1.492E-04	3.184

MF2S2, mixed culture fermentation (*C. railenensis* and *S. cerevisiae*); S2, pure culture fermentation (*S. cerevisiae*). Similarity indicates that the matching degree between the substance detected and the substance in the standard library; the value range is from 0 to 1,000, with a higher value indicating a better match. RT represents the chromatographic retention time of the substance. Mass indicates the mass-to-charge ratio of substance characteristic ions. Values of MF2S2 and S2 indicates the mean of the relative quantitative values of the volatile aroma compounds ± the standard deviation values (VOC GC peak area/IS GC peak area). VIP is variable importance in the projection of the first principal component in OPLS-DA model. FC (fold change) indicates the multiple relationship of the substance between the two groups; when FC > 1, the substance is marked by “^*^”. Underline indicates FC > 1 value.

#### 3.3.1. Esters

Esters are considered particularly important for wine flavor, mainly providing desirable fruity notes (Swiegers et al., 2005; Dzialo et al., 2017), which can influence the aroma of icewine through direct and complex synergistic interactions. A total of 20 esters were identified and screened in the pure and mixed culture fermented icewines, including 7 acetate esters and 13 ethyl esters (Table 4). Except for ethyl valerate, the other esters in the icewine of S2 were higher than those in the icewine of MF2S2 (FC values < 1). The highest FC value is 1.114 (ethyl valerate), and the lowest one is 0.574 (diethyl succinate) in 20 esters.

#### 3.3.2. Alcohols

Alcohols are a diverse group of compounds that can be derived from grapes and fermentation (Huang et al., 2018). A total of 15 alcohols were identified and screened in the icewines, including 5 lower alcohols (<6 carbons), 6 higher alcohols (≥6 carbons), 2 aromatic alcohols, and 2 other alcohols (Table 4). The five lower alcohols in the icewine of S2 were all higher than those in the icewine of MF2S2 (FC values <1, from 0.841 to 0.912). In terms of higher alcohols, 3-octanol and 2-ethylhexanol in the icewine of MF2S2 were both higher than those in the icewine of S2, and their FC values were 1.094 and 1.196, respectively. Conversely, the FC values of 1-hexanol, 1-nonanol, 1-octanol, and 1-octen-3-ol are <1 (from 0.650 to 0.946).

Benzyl alcohol, 2-phenylethanol, and 3-methylthiopropanol in the icewine of mixed culture fermentation were lower than those in the icewine of control fermentation, and their FC values were 0.879, 0.898, and 0.818, respectively. Moreover, 2-furanmethanol was the highest FC value (3.388) among the 52 volatile aroma compounds.

#### 3.3.3. Terpenes and others

Terpenes have an important effect on wine aroma and are responsible for the characteristic floral and fruity aroma (Englezos et al., 2018; Hong et al., 2021). A total of 7 terpenes were detected in the icewines, and the highest FC value is 1.745 (β-damascenone), and the lowest one is 0.551 (Linalool), as shown in Table 4.

Fatty acids, ketones, aldehydes, and furans are also indispensable compounds of wine aroma. As shown in Table 4, there are 3 fatty acids, 4 ketones, and 3 aldehydes in others. The FC values of hexanoic acid and octanoic acid are more than 1 (1.172 and 1.309). About ketones, the relative contents of acetoin with butter and creamy aroma (Francis and Newton, 2005) and 2-acetylfuran with nutty and sweet caramel-like aroma (www.chemsrc.com) were higher in the mixed fermented icewine than in the pure fermented one, and their FC values were 1.259 and 2.443, respectively. On the contrary, 2-octanone and acetophenone in the icewine of mixed culture fermentation were lower than those in the icewine of control fermentation, and 2-octanone has the lowest FC value (0.450) among the 52 volatile aroma compounds. In the three aldehydes, the FC values of both benzaldehyde and 5-methyl furfural are more than one, while the FC value of undecan-4-olide is 0.949.

### 3.4. Sensory evaluation of icewine

**Figure 5:**
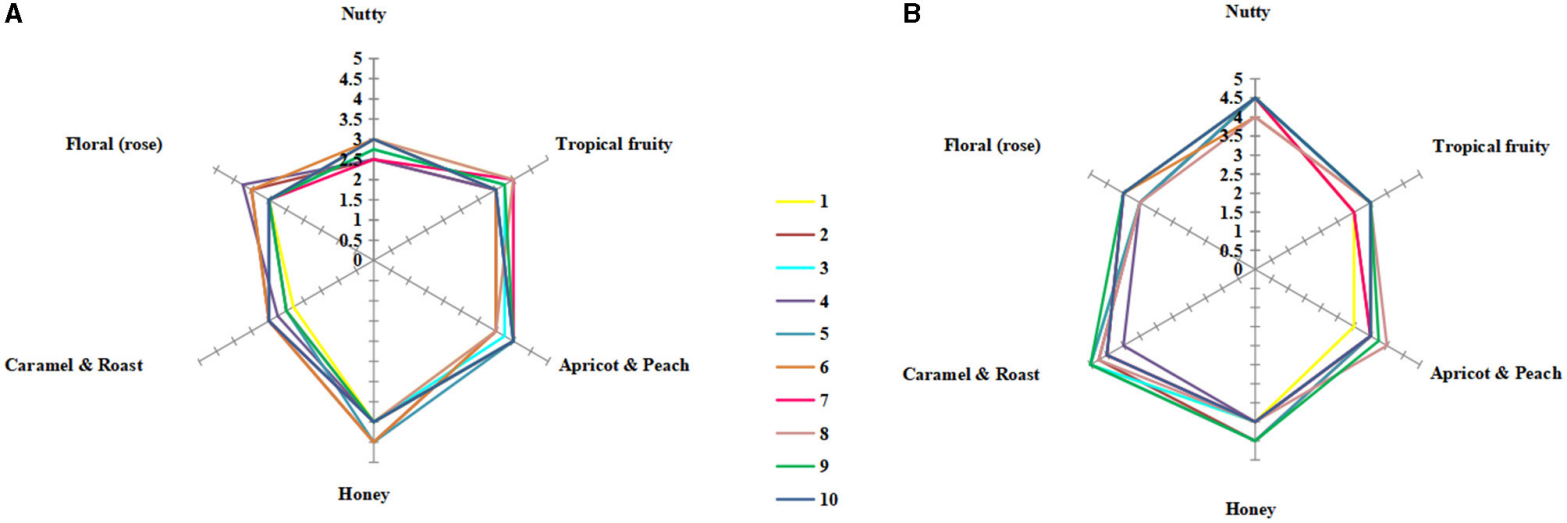
Sensory radar charts of icewine aroma of S2 **(A)** and MF2S2 **(B)**. 1–10 denotes the number of tasters selected. The maximum score for each aspect of aroma is 5, the higher the score, the stronger the aroma: 0–1 means very weak, 1–2 means weak, 2–3 means medium, 3–4 means strong, and 4–5 means very strong. Each final score is twice the mean of tasting scores.

The sensory evaluation of pure and mixed culture fermented icewines was carried out. The main aromas of Vidal blanc icewine were nut, honey, caramel, rose, tropical fruit, and apricot (Huang et al., 2018). Therefore, six aspects of aromas were graded, and the final scores of the icewines are shown in Figures 5A, B.

The aroma profiles of the icewines under different fermentation strategies were clearly different. The aroma profile of the mixed culture fermented icewine was characterized by caramel and roasted and honey aromas, while the pure culture fermented icewine expressed fruity and honey aromas. Furthermore, the overall sensory evaluations of both icewines are as follows: colors were both light golden yellow; aroma intensity, taste intensity, smoothness, and viscosity of the mixed culture fermented icewine were stronger than those of the pure culture fermented icewine; they were both harmony and balance. Therefore, *C. railenensis* can change the aroma profile and sensory performance of icewine by participating in the fermentation of *S. cerevisiae*.

## 4. Discussion

In this study, we showed for the first time the growth dynamic changes of *C. railenensis* and the effect of mixed culture of *C. railenensis* and *S. cerevisiae* during fermentation on the aromatic profile of Vidal blanc icewine, and pure *S. cerevisiae* fermentation was used as the control. During both types of fermentation, *S. cerevisiae* increased first and then declined because most of the sugars in the fermenting medium were consumed (Englezos et al., 2019). During the mixed culture fermentation, the highest cell population of *S. cerevisiae* was slightly lower than that in the pure culture fermentation, and this may be due to competition for nutrient consumption between *S. cerevisiae* and non*-Saccharomyces* yeasts, which was caused by cell-to-cell contact mechanisms (Fleet, 2003). *C. railenensis* increased after inoculating *S. cerevisiae* from day 2 to day 4, and this may be because *S. cerevisiae* promoted the growth of *C. railenensis*; further research would be needed to illuminate the interaction of these two species during fermentation. Then, *C. railenensis* disappeared after 14 days, which was because the ethanol content accumulated through fermentation had reached or exceeded the ethanol tolerance of *C. railenensis* (>4 and <8%, v/v; Hong et al., 2019).

The basic chemical composition of icewine was affected by the inoculation of *C. railenensis* during fermentation. In the early stages of fermentation, lower levels of CO_2_ production and ethanol contents in the mixed culture fermented icewine suggested that the ability for alcoholic fermentation of *C. railenensis* was lower than that in *S. cerevisiae*; *C. railenensis* had a negative influence on the alcohol fermentation performance of *S. cerevisiae* at the initial stage of inoculation with *S. cerevisiae*, and this may be because of competition for sugar consumption between *S. cerevisiae* and *C. railenensis*. At the end of fermentation, lower CO_2_ productions and ethanol contents in the mixed culture fermented icewine showed that *C. railenensis* can have the effect of reducing ethanol content during icewine fermentation. Many studies have shown that non-*Saccharomyces* yeasts (such as *C. zemplinina*) have the effect of reducing ethanol content in wine fermentation (Giaramida et al., 2013; Contreras et al., 2014; Englezos et al., 2019). In recent years, consumers have become more interested in wines with lower ethanol concentrations for health and taste reasons (Ballester-Tomás et al., 2017; Li et al., 2022), and higher ethanol concentration has an undesired effect of reducing the complexity of wine sensory properties (Contreras et al., 2014). In terms of glycerol, higher glycerol content in the mixed culture fermented icewines suggested that *C. railenensis* could increase glycerol yield; therefore, the smoothness and viscosity of icewine structure/body perception of mixed culture fermentation should be stronger than those of the control fermentation (Swiegers et al., 2005), and the sensory analysis confirmed this fact. Meanwhile, glycerol contents of the icewines were higher than those in dry wines. This was because the high sugar content in ice grapes must cause the expression of yeast glycerol-3-phosphate dehydrogenase (GPD1) to be upregulated, resulting in higher glycerol yields during fermentation (Pigeau and Inglis, 2005). Glycerol concentration in dry wine is generally 4–10 g/L (Heit et al., 2018), and more glycerol is considered to improve wine quality. Furthermore, acetic acid was one of the most important basic chemical compositions affecting the quality of icewine compared with the control fermentation, the acetic acid content produced by mixed culture fermentation was lower, this demonstrated that *C. railenensis* had the effect of reducing acetic acid content.

In terms of esters, compared with the pure culture fermented icewine, the icewine of mixed culture fermentation produced lower levels of esters, indicating that *C. railenensis* can reduce multiple esters and their flavors. This is similar to the result by Van Wyk et al. (2020), who stated that the involvement of *C. railenensis* resulted in a dramatic reduction in ester content (such as isoamyl acetate, ethyl butyrate, and ethyl hexanoate) in mixed culture fermented Muscaris wine, but ethyl valerate was not detected in their study. The result may be due to the low ester production capacity of *C. railenensis* initially inoculated, or/and *C. railenensis* negatively affected the formation of esters by *S. cerevisiae* during mixed culture fermentation of icewine. The detailed mechanisms still need to be further studied at metabolites and transcriptional levels.

Ethyl esters are formed by an enzyme-catalyzed condensation reaction between ethanol and acyl-CoA during lipid biosynthesis (Saerens et al., 2010). Ethyl valerate is a major ester in Vidal blanc icewine from the Huanren region, China, which has pleasant fruity aromas such as “apple” and “grape” (Ma et al., 2017; Huang et al., 2018). Ethyl acetate, which is a compound responsible for wine deterioration, is lower in the mixed fermented icewine; this was consistent with the result by Englezos et al. (2018), who pointed out that the esters in Chardonnay and Muscat wines of mixed culture fermentation (*C. zemplinina* + *S. cerevisiae*, 48 h) were significantly lower than this aroma family in pure fermented wines. Acetate esters are formed by the condensation of higher alcohols with acetyl-CoA, which are catalyzed by ATF1 and ATF2 of alcohol acyl-transferase (AAT) genes in yeast cells (Peddie, 1990), and they are considered to have a greater effect on the perceived aroma than ethyl esters (Dzialo et al., 2017).

As far as alcohols are concerned, the lower alcohols in the mixed culture fermented icewine were lower than those in the icewine of control fermentation. Isoamyl alcohol is produced by the deamination and decarboxylation reactions from leucine (Leu) during fermentation (Molina et al., 2009), and isobutanol is synthesized in yeast cells *via* the valine (Val) degradation pathway (Dzialo et al., 2017); they could negatively contribute to wine quality because of their herbaceous notes (Englezos et al., 2018). Moreover, 2-methylbutan-1-ol is derived from isoleucine (Ile) catabolism through the Ehrlich pathway (Hazelwood et al., 2008), and 1-butanol and 1-pentanol are often associated with unpleasant tastes, such as bitterness.

Regarding higher alcohols, except for 3-octanol and 2-ethylhexanol, the other higher alcohols in the mixed culture fermented icewine were also lower than those in the icewine of control fermentation. 1-Octen-3-ol, which has a “mushroom” flavor, has been detected in Vidal blanc icewine from the Huanren region, China (Ma et al., 2017; Zhang et al., 2018). 1-Hexanol has a vegetable and herbaceous odor, which negatively affects wine aroma (Englezos et al., 2018). In brief, *C. railenensis* can reduce these lower and higher alcohols and their unpleasant notes but can increase 3-octanol and 2-ethylhexanol contents and their favorable flavors, such as “pine nuts” notes and sweet and slightly floral notes (www.chemsrc.com).

Furthermore, the result showed that *C. railenensis* could reduce the contents of benzyl alcohol, 2-phenylethanol, and 3-methylthiopropanol and their odors. Benzyl alcohol can contribute a slightly sweet fruit odor to wine. 2-Phenylethanol, which is synthesized by the transamination of L-phenylalanine (Phe) *via* the Ehrlich pathway, has a rose-like odor (Swiegers et al., 2005). 3-Methylthiopropanol is derived from methionine (Met) catabolism through the Ehrlich pathway (Hazelwood et al., 2008), and it has a strong onion and meat odor, which negatively influences wine quality.

Notably, isoamyl alcohol, isobutanol, 2-methylbutan-1-ol, 2-phenylethanol, and 3-methylthiopropanol are derived from their corresponding amino acid catabolism *via* the Ehrlich pathway, which is caused by living yeast cells during fermentation (Hazelwood et al., 2008). These alcohols in the icewine of mixed culture fermentation were lower than those in the pure culture fermented icewine, suggesting that *C. railenensis* with a lower ability to produce these alcohols could negatively influence the formation of products of the Ehrlich pathway by *S. cerevisiae* in mixed culture fermentation, and the detailed mechanism would be studied in the future. Interestingly, 2-furanmethanol presented at a significantly higher level in the mixed culture fermented icewine and has creamy and caramel aromas (Lee et al., 2010). It could contribute to wine with coffee flavor through synergistic action with furfural in wine (Naudé and Rohwer, 2013).

Terpenes usually exist in the form of non-volatile and non-aromatic compounds during wine fermentation, which is complexed with glycosides and can be released by hydrolases, and different expression levels and activities of these hydrolases have been demonstrated in yeast species in previous studies (Fernández et al., 2000; Hong et al., 2019). Van Wyk et al. (2020) pointed out that *C. railenensis* could increase the content of β-damascenone during mixed culture fermentation, which was consistent with the result of this study, but their study just focused on general wine of mixed culture fermentation. β-damascenone is indirectly produced from β-carotene *via* neoxanthin in wine by oxidative cleavage, followed by enzymatic reduction and acid catalysis reactions (Timmins et al., 2020). The increased content of β-damascenone may be attributed to the stronger ability of *C. railenensis* to release (or convert) grape varietal compounds than *S. cerevisiae*, as well as the higher expression levels and activities of related enzymes. Moreover, β-damascenone, which has an intense rose fragrance, honey flavor, and apricot peach aromas, has been reported as a key odorant in Vidal blanc icewine profiles (Ma et al., 2017; Huang et al., 2018; Tang et al., 2021). Notably, the odor threshold of β-damascenone is very low (0.05 g/L), and small changes in the concentration can markedly influence sensory evaluations of wine (Francis and Newton, 2005). Therefore, *C. railenensis* in mixed culture fermentation can increase β-damascenone and its pleasant aroma.

Hexanoic acid and octanoic acid are medium-chain fatty acids that can give the wine a cheesy and fatty odor (Francis and Newton, 2005; Hong et al., 2021); and they are in the mixed culture fermented icewine were higher levels than those in the icewine of control fermentation. However, this was inconsistent with the result by Van Wyk et al. (2020), who stated that hexanoic acid and octanoic acid in wines of mixed culture fermentation were not higher than those in pure fermented wines.

In addition, the result showed that *C. railenensis* could increase 2-acetylfuran, benzaldehyde, and 5-methyl furfural contents and their pleasant aromas. 2-Acetylfuran can contribute to a nutty and sweet caramel-like aroma to wine. Benzaldehyde is described as having fruity, nutty, and caramel-like aromas (Xiao et al., 2015). 5-Methyl furfural, which derives from hexoses present in cellulose, can contribute caramel-like and toasted almond aromas to wine (Dumitriu et al., 2019). Correspondingly, the distinct nutty and caramel aroma in the mixed culture fermented icewine was found in the results of the sensory analysis.

## 5. Conclusion

The effect of sequential inoculation of indigenous *C. railenensis* and *S. cerevisiae* on the alcoholic fermentation behavior and the chemical and aromatic characteristics of Vidal blanc icewine was unveiled for the first time. The results indicated that *C. railenensis* in the mixed culture fermentation increased glycerol yield and reduced acetic acid yield in the basic chemical parameters, and the mixed culture fermented icewine with indigenous *C. railenensis* and *S. cerevisiae* had different aroma characteristics compared with the icewine of *S. cerevisiae* fermentation. For the detected volatile aroma compounds, *C. railenensis* reduced some metabolites (such as lower alcohols, 1-hexanol, and 3-methylthiopropanol) and their unpleasant notes and increased the production of most of the desired volatile aroma compounds (such as β-damascenone, 2-furanmethanol, and 5-methyl furfural) associated with rose, honey, nut, and caramel characteristics. Collectively, the results confirmed that *C. railenensis*, with positive enological properties, has the possibility of application in icewine production. Further investigations should be conducted on the application security and metabolic mechanisms of *C. railenensis*.

## Data availability statement

The original contributions presented in the study are included in the article/supplementary material, further inquiries can be directed to the corresponding author.

## Author contributions

JL designed the experiments, analyzed the experimental data, and wrote the manuscript. MH conducted the experiments. All authors contributed to the article and approved the submitted version.
